# Stepped Care Model for Developing Pathways of Screening, Referral, and Brief Intervention for Depression in Pregnancy: A Mixed-Method Study from Development Phase

**Published:** 2022-03-30

**Authors:** Prerna Kukreti, Ramdas Ransing, Pracheth Raghuveer, Mahesh Mahdevaiah, Smita N Deshpande, Dinesh Kataria, Manju Puri, Omsai Ramesh Vallamkonda, Sumit Rana, Harish K Pemde, Reena Yadav, Shilpi Nain, Shiv Prasad, Bhavuk Garg

**Affiliations:** Department of Psychiatry, Lady Hardinge Medical College, New Delhi,; Department of Psychiatry, Lady Hardinge Medical College, New Delhi,; Department of Psychiatry, Lady Hardinge Medical College, New Delhi,; Department of Psychiatry, Lady Hardinge Medical College, New Delhi,; Department of Psychiatry, Lady Hardinge Medical College, New Delhi,; Department of Psychiatry, Lady Hardinge Medical College, New Delhi,; Department of Psychiatry, Lady Hardinge Medical College, New Delhi,; 1 Department of Psychiatry, B.K.L. Walawalkar Rural Medical College, Sawarda, Maharashtra,; 2 Department of Community Medicine, Kasturba Medical College, Mangalore, Manipal Academy of Higher Education, Manipal,; 3 Department of Psychiatry, Dharwad Institute of Mental Health and Neurosciences, Dharwad, Karnataka, India; 4 Department of Psychiatry, Health Sciences, Bengaluru,; 5 Department of Obstetrics and Gynaecology, Lady Hardinge Medical College, New Delhi,

**Keywords:** Brief intervention for depression in pregnancy, formative research, perinatal depression, stepped-care model

## Abstract

**Background:**

Depression in pregnancy affects nearly one in five women in low- and middle-income countries and is associated with adverse obstetric and neonatal outcome. Burden of morbidity is high, but specialized mental health resources are meager. Effective low intensity psychosocial interventions hold promise to fill the treatment gap for maternal depression. In this paper, we aim to describe the process of development of a stepped care model incorporating screening, providing brief intervention, and referral pathways developed for managing depression in pregnancy in antenatal care health facilities in India.

**Methodology:**

Using complex intervention development and evaluation method of Medical Research Council, United Kingdom, we searched evidence-based strategies from preexisting manuals, conducted formative research for need assessment and stakeholder engagement, and developed the intervention following an expert review panel. We conducted pilot testing to assess the feasibility and acceptability of intervention supplemented by three focused group discussions.

**Results:**

Manual review identified psychoeducation, empathetic listening, behavior activation, and supportive counseling as important elements. Need assessment revealed huge gap in perinatal mental health knowledge. Nearly 92% of total 272 perinatal women had poor awareness and 35%–70% of total 62 health-care providers had poor knowledge. In qualitative interview, women reported depressive symptoms as a normal part of pregnancy and had poor help seeking, behavior symptoms of depression were more prominent. A stepped care algorithm was developed for screening all expectant mothers in each trimester for depression using Patient Health Questionnaire-9 (PHQ-9). Women with PHQ-9 score >19 or reporting self-harm ideation were urgently referred to psychiatrist. Women with PHQ-9 score 5–19 were given brief intervention for depression in pregnancy intervention by antenatal nurse. The intervention developed consists of three sessions of psychoeducation, relaxation exercise, and mental health promotion, each lasting 20 min and at gap of 2 weeks each. Service providers and mothers reported good acceptability of psychosocial intervention and reported satisfaction with content and delivery of intervention.

**Conclusion:**

Low intensity brief psychosocial interventions can be adapted for implementation if relevant stakeholders are engaged at each step right from development of such as screening, intervention pathway to delivery, and effectiveness study.

## Introduction

Globally, depression is the leading cause of mental health disability, and the magnitude is increasing with successive year.^[1]^ Perinatal period due to inherent biological, psychological, and social transitions make women more vulnerable to develop depressive and anxiety disorders. Depression is the second most common perinatal mental health disorder affecting up to one in ten women in high-income countries and nearly one in five women in low- and middle-income countries (LAMIC).^[2]^ Research indicates that untreated maternal depression is associated with detrimental effects on maternal physical health during pregnancy,^[3]^ obstetric outcome,^[4]^ infant outcome,^[5]^ and early child development.^[6]^

Prevalence rates of maternal depression in the past decade have increased not only due to increased risk factors such as loss of social rubric of the joint family system for childcare support, more mothers being employed with variable maternity leave benefits, rise in domestic violence cases, and increased global conflictual situation but also due to positive changes such as increased awareness, advocacy movement, and isolated research or program-based active screening for maternal mental health in a few countries.^[7]^ Demand for addressing maternal depression has increased considerably, but mental health professionals available to screen and provide effective intervention remain meager especially in LAMIC. Literature has emerged in favor of effective low-intensity psychosocial intervention models for addressing maternal depression and has shown improvement in perinatal depression, health-care seeking, breastfeeding as well as physical growth and risk of infection in infants.^[8,9]^ Specifically, stepped care models utilizing existing antenatal health-care staff or peers for screening maternal depression and providing brief intervention have shown promising results across South Asian countries.^[8,9]^

With the promises shown in research setting for these programs, challenges have also emerged concerning feasibility, scalability, sustainability, and acceptance for real-time integration of these models in existing maternal child health programs.^[9]^ For effectiveness in LAMIC, such models need to be simple enough for lay health-care workers to use and adapt in different health care and cultural settings.^[10]^

### Study aims

The purpose of this study is to describe the process of developing and implementing a stepped care model incorporating screening, providing brief intervention and referral pathways developed for managing depression in pregnancy in antenatal care health facilities in India. This model was developed as a part of multicentric randomized controlled trial (RCT) for developing a brief intervention for depression in pregnancy (BIND-P) in four centers across different parts of India.^[11]^ Medical Research Council, United Kingdom (MRC, UK) proposes four stages for implementation of complex psychosocial interventions: Development, feasibility, evaluation, and implementation.^[12]^ Index study will focus only on development and feasibility aspects only.

## Methodology

### Ethical approval

The study received ethical approval from the Institutional Ethical Committee of all four study sites. Participant recruitment was started only after trial registration in Clinical trial registry of India (CTRI/2018/07/014836). Written informed consent was taken from all participants after explaining them nature and purpose of study. Anonymity of participants was ensured, and due standard treatment was provided to all screen-positive mothers with depression including urgent referral for severe cases. Contact detail of one resource person from each study site was provided to approach to in case of need.

### Setting

The study was conducted in the antenatal outpatient department of the Department of Obstetrics attached to medical colleges across India: Lady Hardinge Medical College (LHMC), Delhi (Northern India); BKL Walwalkar Rural Medical College (BKLWRMC), Ratnagiri, Maharashtra state (Western India), Yenepoya Medical College (YMC) Mangalore and Dharwad Institute of Mental health and Neuroscience (DIMHANS) Karnataka (Southern India). All three sites added diversity in terms of culture and level of healthcare setting. The first three sites were tertiary care centers, and fourth one was a secondary level health-care facility. Study centers also offer a mix of rural (Ratnagiri) as well as urban settings and public health facility (LHMC and DIMHANS) as well as private health facility (BKLWRMC, YMC).

### Study design

A mixed-method study was used; triangulation of methods was done to extract information from multiple sources. Based on guidelines of developing complex interventions, following five steps were followed for intervention development and feasibility assessment:

### Pre stepped care model development

#### Step 1: Literature review

To select evidence-based approaches from literature with proven effectiveness for developing screening, providing brief intervention, and referral pathways for maternal depression.

#### Step 2: Need assessment

Knowledge attitude survey and qualitative interviews with all stakeholders, service users, as well as service providers.

### Designing stepped care model

#### Step 3: Expert consultation review

Consultation review with subject experts involving psychiatrist, psychologist, obstetrician, pediatrician, and public health experts to decide structure and content of intervention.

#### Step 4: Feedback from service users and providers

To determine feasibility and acceptability of stepped care model delivery using qualitative interview with stakeholders.

#### Step 5: Refining the stepped care model

Finalizing the model based on feedback received after expert consultation review.

## Methods and Results

### Pre stepped care model development

#### Step1: Literature review

##### Methods

In India, screening for mental disorders in perinatal period is not incorporated in routine maternal health-care pathways. Thus, utilizing distillation-matching-method principles,^[13]^ search was done for preexisting evidence available regarding screening referral and brief intervention pathways for maternal depression. The aim was to examine individual elements of such interventions or strategies for adaptation in our setting. Electronic databases such as EMBASE, PsycINFO, MEDLINE, CINAHL, and the Cochrane Central Register were searched using keywords “maternal depression, perinatal depression, postnatal or postpartum depression” AND “stepped care model, stratified model, RCT, low-intensity psychosocial intervention, effectiveness trial and clinical trial” from 2007 to 2018. Search was restricted to individual-based psychosocial interventions with structured manuals with proven effectiveness in a large-scale RCT, simple enough to be delivered by nonspecialized health care worker in LAMIC suiting to our resources and cultural context.

##### Results

Evidence review showed most effective interventions utilized components of cognitive behavior therapy, problem-solving therapy, interpersonal therapy, empathetic listening, supportive counseling. We found five programs which either focused on training of healthcare workers or self-help skills available in the public domain, namely; Thinking Healthy Program (THP) manual,^[14]^ Perinatal Mental Health Project (PMHP) manual on basic counseling skills: A guide for health-care workers in maternal care,^[15]^ A Guide to Maternal Mental Health by White Swan Foundation (WSF) for expectant mothers and families,^[16]^ Lifeline basic counseling skills participant manual,^[17]^ Africa Focus on Intervention Research for Mental health, (AFFIRM) perinatal counseling manual.^[18]^ Of theses, WSF manual was a resource guide for family and patient only; PMHP, AFFIRM manuals focused more on training aspect of health-care workers rather than clinical care implementation pathways. THP intervention had shown good effective size but had a long duration of therapy. Hence, elements of relevance for our manuals such as psychoeducation, cognitive restructuring, mother–child relationship, mental health promotion strategies, and involvement of significant family members were distilled from preexisting manuals. Results are tabulated in Table 1.

#### Step 2: Need assessment

##### Survey for knowledge assessment

###### Methods

A cross-sectional survey was carried out for assessing knowledge about maternal depression using a semi-structured proforma based on Perinatal Depression Monitor among consecutively selected 270 perinatal women attending routine antenatal clinic (ANC) or postnatal clinic (PNC) and 62 health-care providers (nursing professionals and medical professional) working in ANC/PNC clinics or wards of two study centers Delhi and Ratnagiri. Details regarding survey methodology have been reported earlier.^[19]^

##### Result

It showed only 8.51% of perinatal women had awareness regarding maternal depression. Among nursing professional surveyed, most (71%) regarded it as normal part of pregnancy, only 45% considered it as biological and only 23% regarded antidepressant medications as a part of treatment for same. Among medical professionals, up to 90% recognized it as a medical problem, 70% considered it as having biological cause needing antidepressant medications. Sociodemographic details have been reported earlier.^[19]^

#### Semi-structured interview

##### Methods

Purposive sampling was done on the following two groups of voluntary participants (perinatal women diagnosed with depressive disorder and nursing professionals working in perinatal area) from two centers Delhi and Ratnagiri.

- *Participants understanding about maternal depression and expectations from screening and intervention strategies*: Focused group discussion (FGD) were carried out in the successive women of 18–40 years of age group visiting antenatal or PNC diagnosed as having depressive disorder by a psychiatrist as per ICD 10, Diagnostic Criteria for Research (World health Organization, 1994)^[20]^ willing to voluntarily participate. Interview was conducted using a prestructured guide regarding their understanding of psychological, social, and cultural aspects of depression, explore thinking-styles of problem-solving, expected determinants in any intervention
- *Nursing professionals understanding about maternal depression, need of screening, self-perceived ability and willingness to deliver intervention*: In-depth interviews were conducted with purposively sampled volunteer nurses working in the antenatal and postnatal OPD. Staff of varying age and experience was recruited to understand the issues involved in delivering a psychological intervention in a hospital setting. We explored the nurse’s daily work profile, their views about psychosocial intervention delivery, feasibility, challenges of working with peripartum women and families, health beliefs, help-seeking behavior and presence of existing resources for dealing with perinatal mental health problems. Interviews allowed free discussion while permitting maximum elaboration of participant response. Issues raised in one interview were used as probes for the next interview
- All interviews followed an iterative process till themes were saturated with no prior fixed sample size. All interviews were recorded, transcribed verbatim, and analyzed as per framework analysis. Two researchers familiarized themselves with the data, codes were generated, and subthemes were generated manually.

##### Results

Overall, 35 mothers with depressive disorders (20 antenatal women, 15 postnatal women) and 12 Nursing professionals were interviewed. Key findings regarding mother’s views are highlighted in Table 2. Most mothers regarded depressive symptoms as either normal physiological change of pregnancy or transient issue of adjusting to motherhood. They did not regard it as an illness. They wanted assessment of mental and physical health integrated in one setting and expressed marked difficulty in visiting mental health services separately due to time constrain and stigma.

Most nursing professionals welcomed brief screening as an essential thing to be incorporated in perinatal care, but most had a concern about intervention is either time consuming or too difficult and expressed the need of training for counseling skills and continued supervision and assistance. Key findings regarding nursing professional’s views are highlighted in Table 3.

#### Step 3: Expert consultation review

##### Methods

To develop an intervention grounded in evidence-based interventions yet culturally well-adapted to and feasible for the Indian population, we established a multidisciplinary team with expertise in several domains. The teams included two clinical psychologist, eight psychiatrists, three obstetricians, one pediatrician, two public health experts, and one psychiatry nursing faculty with considerable experience in mental health conditions and puerperal disorders.

##### Results

A stepped care model was developed taking into account extensive review of literature done for low-intensity psychosocial interventions for peripartum disorders, expert panel and author’s own clinical experience and formative research done in service providers and users in perinatal clinics. The main input for the intervention method was based on the outcome of semi-structured interviews, survey results, and WHO mhGAP Manual, “Thinking Healthy” that included techniques of active listening, collaboration with family, and homework activity added to the routine maternal child care service model.

### Stepped care model

**Figure 1:**
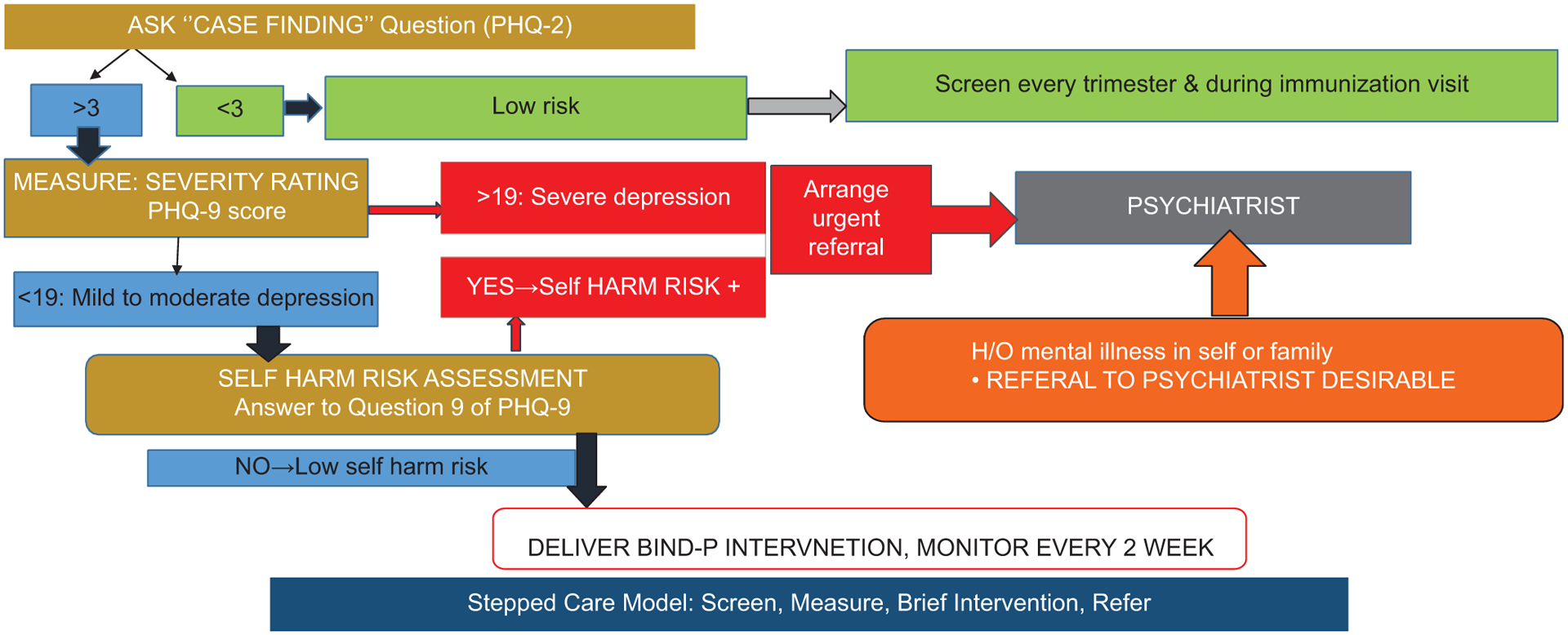
Stepped Care Model for Screening, Risk assessment, Brief Intervention and Referral pathway for maternal depression

A model was developed incorporating the following steps: [Figure1]

#### ASK

Provision of screening of all mothers visiting antenatal OPD by asking questions of short 2 item Patient health questionnaires (PHQ 2). Those who score ≥3, were subjected for further evaluation with PHQ-9.

#### Measure

Screen positive (PHQ-2 score >3) mothers were assessed for severity of depression by using 9-item PHQ 9.

#### Risk assessment

Based on PHQ-9 rating, participants can be divided into two groups.

#### High risk cases

PHQ-9 Score >19 (severe depression) or yes to Question 9 of PHQ-9 assessing self-harm ideation→ Urgent referral to psychiatrist.

*Low to Medium Risk*-→ PHQ-9 score <19 mild to moderately severe case of depression, antenatal nurse to provide brief intervention, BIND-P→ referral to a psychiatrist if no improvement after 6 weeks of BIND-P intervention or progression to severe symptoms during the course of intervention.

(*anybody with past history of psychiatry illness, irrespective of screening tool score should be sent for perinatal care planning to psychiatrist, even if currently in remission).

#### Intervention delivery

BIND-P intervention, a low-intensity psychosocial intervention was developed based on core principals of psychoeducation, empathetic listening, and providing supportive counselling. It can be delivered by a nurse following structured training of 3 days. It consists of four sessions of half an hour each, delivered at gap of 2 weeks. Three sessions focus on psychoeducation and mother–infant bonding, relaxation exercise, and mental health promotion activities. Initially, a fourth session concerning domestic violence was planned but based on stakeholder’s feedback, it was removed. Characteristics of intervention are described in Table 4.

#### Follow-up

All screen negative mothers or mothers who improved on low-intensity psychosocial intervention should continue to be screened in every trimester and during child immunization visits and in case of being found screen positive at any stage, abovementioned protocol should be used

#### Training manual for nurses training

A training module uniform for all sites was developed incorporating the time framework, mode of training, outcome evaluation, and fidelity checks. Resource material was also developed for 3-day module-based capacity building program with inputs from subject experts focusing on:

#### Content of training: The training focussed on

- Identification of depression among pregnant women,
- Using screening and rating instrument PHQ (PHQ-2 and PHQ-9),
- Developing skills such as counseling-based psychoeducation, empathic listening, and befriending specific to the intervention module
- Develop appropriate referral practices for moderate to severe cases.

#### Mode of training

An interactive case-based approach and role play were incorporated in training module for skill building during the training with the provision of *continued training*. Reorientation program with feedback sessions conducted for the trained nurses on a quarterly basis besides the on-site assistance in daily dealing of cases.

*Pre and Post-Test assessments* were carried out to examine the change in knowledge with training and assessing competence by demonstration of skills learned on healthy volunteer actors from research team.

#### Step4: Postintervention development

Feedback from service users and providers.

##### Methods

The fourth step was a qualitative assessment of draft stepped care model and intervention through a pilot study in Delhi. Ten antenatal and ten postnatal women with depression having PHQ-9 <19 were purposively picked and given intervention after informed consent. After 6 weeks of start of pilot trial, FGDs were conducted in service users and providers to ascertain the feasibility, acceptability, and adaptability of intervention content or frequency or modality. Three FGDs were held with a group of 5–6 people each, consisting of nursing professionals, antenatal women, and postnatal women each. An interview guide was created to ask question regarding usefulness of intervention, views about content, any suggestions to improve or any facilitation strategy suggested, any difficulty encountered. All FGDs were recorded, transcribed, and key thematic areas coded and analyzed manually.

##### Results

###### Mothers’ views

All mothers found it acceptable, 83% reported it to be useful and report having learned new perspective about importance of their mental health for maternal–child relationship and infant growth. They appreciated the self-help skills taught as a part of mental health promotion. Sixty-six percent of mothers did not find the last session about domestic violence useful and reported it to be removed from intervention or to be kept only for targeted population reporting it. Mothers also expressed difficulty in coming for fortnightly sessions when their ANC visit is monthly.

###### Service provider’s views

All nursing professional reported no difficulty in using PHQ for screening after training. Nearly 50% suggested to keep intervention sessions limited to two or three to reduce attrition. Nearly 25% expressed concern about extra workload and suggested monetary incentivization as a suggestion to keep staff motivated. Nearly half expressed concern about long-term sustainability of these practices in the absence of its incorporation in programmatic approach and suggested if translation of research can be converted into implementation by making it a mandatory top-down order for inclusion in routine functioning and involving hospital administration.

#### Step 5 finalizing the stepped care model and brief intervention for depression in pregnancy intervention

No changes were suggested in stepped care model. Based on result of pilot study, BIND-P intervention was modified, and last session concerning domestic violence was removed, elements of mother–infant interaction were shifted to the first session of the intervention, and sessions were reduced to three focusing on psychoeducation, relaxation exercise, and mental health promotion. Final result of stepped care model remains same as described in Figure 1. Final version of intervention has been translated from English into Hindi, Marathi, and Kannada.^[21,22]^

## Discussion

Despite years of research, most focus still remains on “postpartum depression” neglecting the fact that of all the disorders surrounding peripartum period more than half have onset antedating the delivery.^[23]^ Furthermore, there have been multiple disjointed efforts of assessing and intervening either in antenatal period or only in postpartum period; however, there is an incumbent need of developing systematic protocols for assessing mothers right from conception for preemptively identifying high-risk mothers in need and continuing the momentum of care postnatally also, preferably up to 6 months to 1 year after delivery.

This paper describes the formative phase of developing one such stepped care model based on MRC, UK framework for development, and evaluation for complex interventions.^[12]^ The model development process is based on a well-established theory of behavior change utilizing the principle of assess, build, create, and deliver. The use of mix methods allowed for need assessment as well as contextualizing the strategy from stakeholder’s perspective and building an intervention with strong theoretical foundation.

The qualitative interview showed mothers are not able to recognize depressive symptoms, and behavioral symptoms were more prominent than cognitive symptoms. It led to the inclusion of behavior activation strategies in BIND-P intervention, similar to THP, and AFFIRM interventions.^[14,18]^

Preliminary service provider’s feedback showed it is possible to incorporate brief screening elements in busy antenatal OPDs, and if session lengths are kept short and number of sessions limited, nursing professionals may incorporate it in their schedule as in BIND-P intervention. Most other manual-based programs have 8–14 sessions of 40to 60 min each.^[14–18]^ It demonstrates that such low-intensity interventions can be implemented to reduce the treatment gap.

BIND-P not only is an intervention but also is a strategic pathway for stepping up care by incorporating universal screening in maternal–child health service delivery, risk stratification and based on risk assessment, referral, or brief intervention delivery within obstetric setting. It is simple enough for any Accredited Social Health Activist or Auxiliary Nurse Midwife to deliver following due training. BIND-P intervention *per* SE is a simple psychosocial intervention of three sessions of 20–30 min each to be given at a gap of fortnight and needs a formal training and needs competence assessment posttraining.

Concern for this or any such strategic program is need of continued supervision and regular retraining and handholding sessions for health-care provider to ensure fidelity of service delivery and scalability of such interventions. Interventions based on THP manual and AFFIRM manual have also shown similar promises and challenges.

If implemented well, any nursing professional with basic training can be equipped with skills to screen for depression during antenatal visits of mother, manage the mild cases at their own level by psychological intervention and refer moderate or severe cases to mental health expert. It could transform a routine antenatal setting into a one-stop clinic providing holistic services for physical as well as mental health needs.

For increasing the generalizability, effectiveness analysis research is being undertaken at four sites representative of culturally diverse settings of country (northern, central, and southern part of India, taking both rural and urban sites) and simultaneously incorporating all possible health settings of antenatal care, that is, secondary as well as tertiary health care facilities.

### Limitations

Literature review was based on structured review not a systematic review. We restricted our search to LAMIC countries individual-based interventions provided by healthcare workers, we may have missed group based or self-help or peer led innovative models and their inherent elements. Effectiveness trial is running at four centers but index study shared data about pilot testing at Delhi and Ratnagiri centers only. Sample size for postintervention development assessment was small and might have yielded limited response, but it was appropriate for a qualitative formative phase of study. Qualitative analysis was done manually due to nonavailability of access to paid qualitative software. It is also likely speculated that service users and service providers interviewed for the study may not have been able to provide in depth inputs about stepped care pathway or intervention because of the absence of any such screening program in India in routine health-care settings.

## Conclusion and Future Directions

BIND-P stepped care pathway is a systematic path for integration of mental health principles in general health-care settings. Results from the formative phase of the study show engagement with stakeholders during pathway development can give good insights regarding implantation challenges and barriers to effect adaptability. The preliminary result suggests this model is feasible, acceptable and has the potential for scaling it up for integration in reproductive child health programs once effectiveness analysis results of the model implementation are available

## Figures and Tables

**Table 1:** Elements distilled for development of manual following literature review

Intervention program	Focus on	Elements distilled
THP	Universal screening Perinatal MH service delivery Psychoeducation Cognitive restructuring MH promotion	Psychoeducation, cognitive restructuring, mother-child relationship, MH promotion strategies and involvement of significant family members, homework exercise
PMHP	Training of healthcare workers	MH promotion strategies
Guide to maternal MH by WSF	Psychoeducation for expectant mothers and families	Psychoeducation
AFFIRM perinatal counselling	Training of healthcare workers	Psychoeducation, cognitive restructuring
Lifeline basic counselling skills participant manual	Training of healthcare workers	MH promotion strategies, Counselling skills

THP=Thinking healthy program, MH=Mental health, PMHP=Perinatal mental health project, WSF=White swan foundation, AFFIRM=Africa Focus on Intervention Research for Mental health

**Table 2:** Key themes and subthemes from semi-structured interview of peripartum women with depression

Themes	Subthemes
Awareness about depressive symptoms in perinatal period	Denial of MH issues in general or depressive symptoms More focus on physical symptoms during pregnancy Main concern only child well being Normalization of depressive symptoms as part of pregnancy Irritability, tearfulness more common presentations than cognitive aspects of depression
MH help currently available	Reliance on informal social support Reliance on self
Desirable attribute of perinatal MH services	MH needs not sufficiently met Outreach services near home were considered important Peer support was considered most important Educational based activities for physical and MH required Additional services should be integrated with mother child health services Regarded counselling or psychological intervention as important to reduce distress but did not recognize it as part of treatment Skeptical about going to any clinician other than obstetric health facility for any treatment Want clinic-based services, concern about disclosing sensitive issues in home surroundings Want an empathetic listener and preferably a female therapist
Barriers to health seeking	MH professionals considered ‘Pagalo ka dr’ Considered it time consuming to visit two specialists (obstetrician and psychiatrist) Family doesn’t allow frequent or long visits during pregnancy Self-perceived stigma about being labelled mad

MH=Mental health

**Table 3:** Key themes and subthemes from semi-structured interview of nursing professionals regarding maternal depression

Themes	Subthemes
Regarded screening as essential	Expressed personal and professional experiences and regarded screening to be mandatory
Feeling overburdened	Antenatal and postnatal areas nurses are burdened with usual work and considered this as an extra work
Motivation	Few staff with personal motivation and young nursing staff an interns were more willing for delivering intervention
Need of training for skill development	Staff felt need of additional training for skill of counseling Reported need of a resource person to be available for oversight
Concern about content of training program	Wanted it to be short in time to get incorporated in crowded OPDs Wanted it to be of lesser number of sessions 2–4 Wanted it to be simple and pictorial for being able to convey illiterate mothers too Wanted some handbook or flipbook for keeping with them to have cues about session content

OPDs=Outpatient departments

**Table 4:** Characteristic of brief intervention for depression in pregnancy intervention manual

InterventionCharacteristic	Description
Theoretical basis	Based on core principles of imparting psychoeducation, empathetic listening and providing supportive counselling
Delivering agent	Nurse with diploma in nursing or ANM
Structure of intervention	Three sessions: Psychoeducation and mother-infant bonding, relaxation exercise and health promotion
Areas covered besides treatment for depression	Mother’s mood and well-being; mother’s relationship with infant and other family members
Training manual	Structured manual with step-by-step instructions for conducting each session
Training sessions; role-plays and discussions	Training based on 3-day training workshop followed by 1-day refresher after 4 months; includes training video with role-plays and discussions
Supervision	Fidelity check: Weekly to fortnightly supervision of half-day sessions in groups of 2–5; discussing problems and “brain-storming” for solutions

ANM=Auxiliary nurse midwife
